# Feasibility of optimizing intensity‐modulated radiation therapy plans based on measured mucosal dose adjacent to dental fillings and toxicity outcomes

**DOI:** 10.1002/acm2.12407

**Published:** 2018-07-09

**Authors:** Seung Won Seol, Sonya Aggarwal, Rie von Eyben, Ziwei Wang, Cato Chan, Carmen Say, Lei Xing, Wendy Hara, Yong Yang, Quynh Thu Le

**Affiliations:** ^1^ Department of Internal Medicine Icahn School of Medicine at Mount Sinai (VA) Bronx NY USA; ^2^ Department of Radiation Oncology Stanford University School of Medicine Stanford CA USA; ^3^ San Diego School of Medicine University of California San Diego CA USA; ^4^ Keck School of Medicine of USC Los Angeles CA USA

**Keywords:** dental filling, head and neck cancer, IMRT, oral mucositis

## Abstract

We prospectively investigated the feasibility of IMRT treatment plan optimization based on dosimeter measurements of lateral tongue mucosal dose adjacent to the dental fillings and evaluated dose‐toxicity relationship and factors affecting oral mucositis (OM) in head and neck cancer patients. Twenty‐nine head and neck cancer patients with metallic dental fillings who were scheduled to undergo fractionated external beam radiation therapy (RT) ± chemotherapy were enrolled. The lateral tongue dose was measured and if the calculated dose for the entire treatment was ≥35 Gy, a re‐plan was generated to reduce the lateral tongue mucosal dose. OM was graded weekly according to Common Terminology Criteria for Adverse Events version 4.0 and the patients completed the Oral Mucositis Weekly Questionnaire‐Head and Neck Cancer. The result showed that it was not feasible to optimize the IMRT plan based on measured tongue dose in most of the patients who needed re‐plan as re‐planning compromised the target coverage in 60% of these patients. The duration of grade (Gr) 2 OM was correlated with measured lateral tongue dose (*P* = 0.050). Concurrent cetuximab was significantly associated with faster onset of Gr2 OM than concurrent cisplatin (*P* = 0.006) and with longer duration of OM (*P* = 0.041) compared to concurrent cisplatin or IMRT‐alone. The pattern of reported pain over time was significantly different for each treatment type (RT and cetuximab, RT and cisplatin and RT‐alone) and depending on the dose level (*P* = 0.006). In conclusion, optimizing the IMRT plan based on measured lateral tongue dose was not feasible. Measured lateral tongue dose was significantly correlated with longer duration of OM ≥Gr2, and concurrent cetuximab was associated with earlier onset and longer duration of OM ≥Gr2.

## 1 INTRODUCTION

Compared to conventional radiation therapy (RT), intensity‐modulated radiation therapy (IMRT) offers highly conformal radiation dose distribution, reducing xerostomia and improving quality of life in head and neck cancer (HNC) patients.^1^, ^2^ However, IMRT plans apply multiple radiation fields and can increase total irradiated volume when compared to less conformal radiation techniques.^3^ Several studies have shown that increased irradiated volume of oral cavity from IMRT can alter the expected toxicity of the treatment such as the rate of acute OM.^3^, ^4^ Narayan et al.^5^ have shown that if the estimated mean dose to the buccal mucosa (as measured by dosimeters) was kept ≤32 Gy over the course of the radiation, only mild mucositis was noticed; however, if the dose exceeded 39 Gy, most patients developed grade (Gr) 2 OM. Dental filling is a factor that can exacerbate this toxicity by inducing radiation scatter and dose perturbation to adjacent mucosa. Moreover, streaking artifact on the computed tomography (CT) scan caused by dental fillings can further exacerbate OM by hindering accurate dose calculation.^6^, ^7^ A large fraction of HNC patients harbor dental fillings at the time of IMRT. Often, patients have persistent slow healing ulcers on the lateral tongue abutting these fillings for weeks to months after therapy, and it has been presumed that dental fillings contribute to these complications. Despite a rapid increase in the use of IMRT and techniques to reduce such effects, the relationship between the dose distribution and toxicity in this group of patients has not been well studied. We sought to understand the relationship and identify associated factors to help reduce OM by means of precautions such as treatment planning intervention.

In this study, we prospectively investigated the feasibility of IMRT treatment plan optimization based on dosimeter measurements of lateral tongue mucosal dose adjacent to the dental fillings. We hypothesized that modulation of an IMRT plan to reduce the dose to adjacent normal mucosa surrounding the dental fillings would decrease the severity and duration of radiation‐related mucositis. We used optically stimulated luminescence (OSL) to measure the lateral tongue dose adjacent to the dental fillings in a standard IMRT plan and generated IMRT re‐plan based on the measured mucosal dose. The main objectives of this study is first, to determine the feasibility of modifying the mucosal dose during treatment by generating IMRT re‐plan based on the measured mucosal dose and second, to evaluate the dose‐toxicity relationship of IMRT treatment and the factors associated with severity of OM in HNC patient with dental fillings.

## 2 METHODS

### 2.A Patient eligibility

Between July 2011 and October 2013, HNC patients with metallic dental fillings who were scheduled to undergo a course of fractionated external beam RT ± chemotherapy at Stanford University were enrolled. Other inclusion criteria were age ≥18, planned radiation dose to the tumor ≥60 Gy at 1.8–2.2 Gy/fx and the ability to understand and sign a written informed consent document. This prospective study was approved by the institutional review board of Stanford University.

### 2.B Radiotherapy

All patients underwent CT simulation ± a positron emission tomography scan using ≤3 mm slices for IMRT planning. Thermoplastic mask extending from the cranium to below the mandible and a customized Accuform headholder were used to immobilize the patient. A standard IMRT plan was generated and patients were treated with megavoltage radiation over a course of ≥6 weeks with a planned tumor dose of ≥60 Gy. The areas of CT artifacts were contoured and filled with CT number of zero. The area of lateral tongue that corresponds to the location of the OSL were defined as right or left lateral tongue, and the mean doses of the defined volumes of right and left lateral tongue were reported as calculated mucosal dose (vs. measured mucosal dose).

### 2.C Dosimeter measurement and optimized IMRT plan

**Figure 1:**
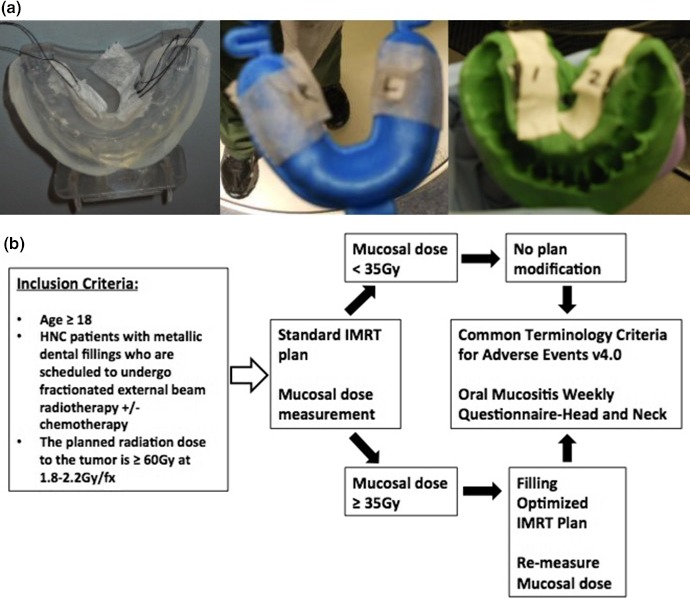
(a) Distribution of dosimeters affixed to mouthpieces used for dose measurement, (b) Study schema.

Measurement of the lateral tongue mucosal dose was performed using OSL dosimeters, which were taped on the lingual side of the lower mouth‐bite (Fig. 1[a]). The OSL was placed on the first day of RT in the 2 mucosal sites: lateral tongue on the left and right sides adjacent to dental fillings. The OSL was developed and measured on the same day. The measured dose per fraction was converted to the total dose for the entire treatment. If the measured total dose for both sides were <35 Gy over the course of the radiation, patients continued through RT using the initial IMRT plan for the entire course. If any of the OSL measurement was ≥35 Gy, IMRT re‐plan was generated to decrease RT dose to that location (Fig. 1[b]). The plan was deemed acceptable and implemented only if it did not compromise tumor coverage and/or increase the dose to the rest of the oral mucosa or spared parotid glands. Using OSL dosimeters, we measured the dose to both lateral tongue sites with the optimized IMRT re‐plan to verify that the measured dose was similar to the revised planned dose.

### 2.D Toxicity and quality of life (QOL) evaluation

Adverse events were graded according to Common Terminology Criteria for Adverse Events (CTCAE) v4.0. Medical doctor performed evaluation and grading of clinical and functional mucositis for the measured site weekly during the RT and biweekly after completion of RT until OM was <Gr2. Patients completed the Oral Mucositis Weekly Questionnaire‐Head and Neck cancer (OMWQ‐HN) during and after treatment until OM was <Gr2.

### 2.E Statistics

The time‐to‐onset of mucositis was analyzed using a Kaplan‐Meier methodology for categorical predictors and a Cox proportional hazards regression model for continuous predictors. The duration of mucositis was analyzed using a mixed effects model to allow for the within‐patient correlation as the time was measured separately for each sides of the patients mouth. All OMWQ‐HN outcomes were analyzed using a mixed effects model to allow for the within‐patient correlation as each patient was measured at multiple time points. Post hoc testing of multiple comparisons was done using a Sidak adjustment. All analyses were performed using SAS 9.4 (SAS Institute, Cary, NC, USA) and all tests were two‐sided with an alpha level of 0.05.

## 3 RESULTS

### 3.A Patient and characteristics and treatment

Patient characteristics are summarized in Table 1. A total of 29 patients were enrolled, 24 men and 5 women. Median age was 61.5 yr (range, 42–82 yr). The primary tumor was located along the oropharynx in 21 patients, oral cavity in seven patients, and nasopharynx in one patient. All patients were treated using IMRT. The median treatment planning system (TPS) dose was 70 Gy (range, 60–70 Gy). Median number of fraction was 33 (range, 30–33). Eleven patients were treated with concurrent cetuximab, 11 with concurrent cisplatin, and seven patients did not receive chemotherapy. Median follow up period was 7 weeks (range, 6–9 weeks).

**Table 1:** Patient characteristics

Characteristic	Number of patients (%)
Gender	
Male	24 (82.8)
Female	5 (17.2)
Age (yr)	
40–60	14 (48.3)
60–80	13 (44.8)
>80	2 (6.9)
Primary tumor site	
Nasopharynx	1 (3.5)
Oral cavity	7 (24.1)
Oropharynx	21 (72.4)
Treatment modality	
Def Cetux + RT	9 (31.0)
Def Cis + RT	9 (31.0)
Def RT alone	1 (3.5)
Postop Cetux + RT	2 (6.9)
Postop Cis + RT	2 (6.9)
Postop RT alone	6 (20.7)
OM ≥ Grade 2	
No	3 (10.3)
Yes	26 (89.7)
Measured tongue dose	
Median	30.1
Range	11.2–64.6

Def, definitive; Cetux, cetuximab; Cis, cisplatin; Postop, post‐operative; RT, Radiotherapy; OM, Oral Mucositis.

### 3.B Dosimetry

Figure 1(a) shows the dosimeters taped on to the different mouthpieces used for dose measurement. Repeated measurements were performed in the first three patients. As shown in Table 2, there was some variability in the measurements, though they were all within 20% of each other and of the calculated dose. Measured doses, TPS calculation and re‐plan information are summarized in Table 3. The median lateral tongue dose was 30.1 Gy (range, 11.2–64.6 Gy). The median percent difference between measured and calculated dose was 14.6% (range: 0%–67.2%). Except for a few outliers, there was general agreement between measured and calculated doses. Forty‐one (70%) out of 58 measurements from each side of lateral tongue mucosa were within 30% differential range of calculated dose, whereas three (5%) measurements showed dose differential greater than 60% of calculated dose for the same site. Possible reasons for disagreement included a large dose gradient present in oral cavity, a dose variation from positioning uncertainties of the dosimeters, radiation scattering caused by metals, and inaccurate calculation on TPS due to streaking artifacts on CT images caused by metals.

**Table 2:** Dosimeter measurement reproducibility over 2–3 consecutive days of treatment

Measure‐ment	Patient #1				Patient #2				Patient #3			
	Measured		Calculated		Measured		Calculated		Measured		Calculated	
	L	R	L	R	L	R	L	R	L	R	L	R
1	50.6	34.1	50.6	32.3	30.5	27.0	34.3	24.2	61.3	50.5	64.4	44.5
2	40.9	28.9			37.0	26.6				57.3	43.5	

L, left; R, right.

**Table 3:** Calculated and measured mucosal dose for both the initial plans and the re‐plans in two locations on the lateral oral tongues, adjacent to the dental fillings in 30 patients with HNC

Pt no.	Tumor site	Tx modality	Measured (Gy)		Calculated (Gy)		Re‐plan	Re‐plan feasible	Re‐plan Calculated (Gy)		Re‐plan Measured (Gy)	
			L	R	L	R			L	R	L	R
1	OP	Def Cetux + RT	42.9	33.8	50.6	32.3	Y	Y	41.7	30.4	ND	ND
2	OC	Postop Cetux + RT	33.8	26.8	34.3	24.2	N					
3	OC	Postop RT alone	59.3	47.2	64.4	44.5	Y	Y	66.0	31.3	63.2	46.0
4	OP	Def Cetux + RT	26.0	26.1	19.0	26.5	N					
5	OC	Postop RT alone	39.9	57.4	24.0	38.6	Y	N^a^				
6	OP	Def Cis + RT	49.6	31.5	60.7	30.7	Y	N^a^				
7	OP	Def Cetux + RT	21.1	53.9	22.5	38.6	Y	Y	24.1	26.7	25.0	26.9
8	OP	Def Cis + RT	18.7	24.1	17.0	14.7	N					
9	OP	Def Cetux + RT	25.3	30.3	28.7	27.5	N					
10	OC	Postop Cis + RT	39.1	22.1	49.4	24.6	N					
11	OP	Def Cis + RT	34.4	34.8	28.1	21.8	N					
12	NP	Def Cis + RT	11.8	27.2	15.7	20.8	N					
13	OP	Def Cetux + RT	32.2	31.1	30.8	35.3	N					
14	OC	Postop RT alone	44.3	62.9	38.5	61.1	Y	N^a^				
15	OP	Def Cetux + RT	30.7	29.4	27.0	24.9	N					
16	OC	Postop Cetux + RT	61.9	61.8	61.9	62.2	Y	N^a^				
17	OP	Postop RT alone	31.2	26.8	29.1	26.4	N					
18	OP	Def Cetux + RT	17.0	17.6	17.0	18.6	N					
19	OP	Def RT alone	31.6	30.1	20.3	18.0	N					
20	OP	Def Cetux + RT	11.4	14.3	27.1	24.3	N					
21	OP	Def Cetux + RT	35.5	44.6	23.4	29.3	Y	Y	20.5	24.3	29.6	39.1
22	OP	Def Cis + RT	34.2	30.7	28.0	23.0	N					
23	OP	Def Cis + RT	29.8	30.1	37.7	33.4	N					
24	OP	Postop Cis + RT	12.7	13.7	19.4	33.5	N					
25	OP	Postop RT alone	11.2	24.5	12.2	28.5	N					
26	OP	Def Cis + RT	17.6	18.4	20.5	29.8	N					
27	OP	Def Cis + RT	18.7	13.0	20.7	16.6	N					
28	OC	Postop RT alone	64.6	57.7	61.5	46.0	Y	N^a^				
29	OP	Def Cis + RT	21.8	38.9	27.8	40.3	Y	N^a^				

Pt, patient; OP, oropharynx; OC, oral cavity; NP, nasopharynx; Tx, treatment; Def, definitive; Cetux, cetuximab; Postop, post‐operative; Cis, cisplatin; RT, radiotherapy; TPS, treatment planning system; L, left; R, right; Y, yes; N, no; ND, not done due to pain issue.

a Re‐plan performed but not implemented due to compromise of PTV coverage.

### 3.C Feasibility of IMRT re‐plan

**Figure 2:**
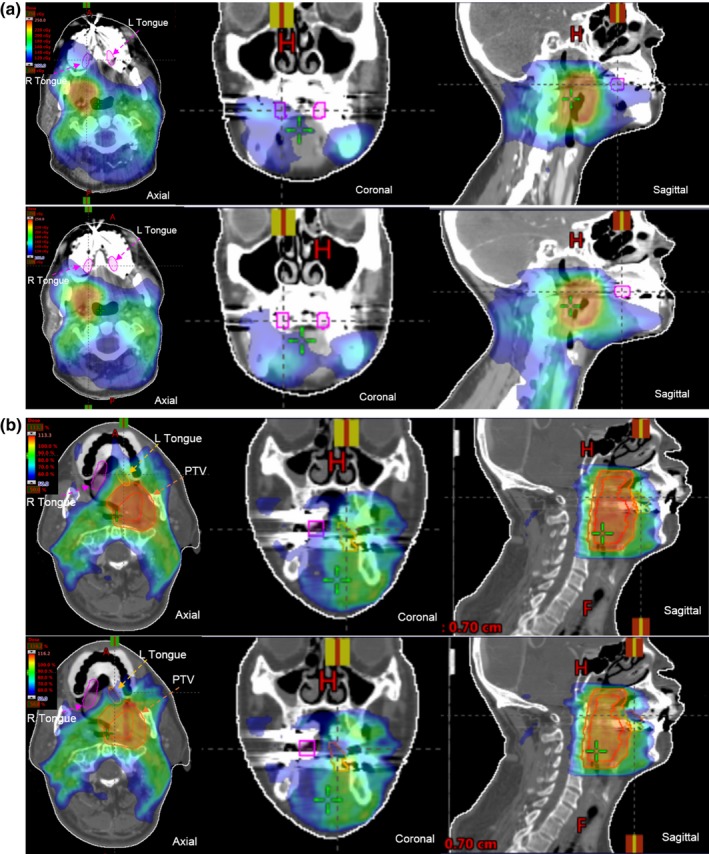
(a) Example (patient no. 7 in Table 3) of dose distributions of initial IMRT plan (upper panel) and feasible IMRT re‐plan (lower panel) (b) Example (patient no. 6 in Table 3) of dose distributions of initial IMRT plan (upper panel) and non‐feasible IMRT re‐plan (lower panel).

As shown in Table 3, the data collected suggested that it is not feasible to optimize the IMRT plan based on measured lateral tongue dose in most of the patients who need re‐plan. For patient no. 10, although the dose on the left lateral tongue was higher than the re‐plan criterion of 35 Gy, the left lateral tongue was very close to planning target volume. Since it was impossible to make an acceptable re‐plan without compromising tumor coverage, the patient no. 10 continued RT using the initial plan for the entire course. Among 10 patients who had attempted re‐plan due to the measured lateral tongue doses exceeding 35 Gy, four patients were treated with re‐plan and six patients were treated with initial plan as re‐planning compromised the target coverage while reducing doses to lateral tongue mucosa. Among the four patients who were treated with re‐plan, less doses to lateral tongue was achieved in two patients. For patient no. 3, although the re‐plan showed reduced radiation dose to right lateral tongue, the measured dose was similar to that of the initial plan. Possible reasons for disagreement here included the combination of large dose gradient across the oral cavity mucosal and the positioning uncertainties of the dosimeters. The lateral tongue dose after re‐plan for patient no. 1 was not measured due to pain and brisk gag reflex. Examples of dose distribution for initial plan vs. feasible re‐plan and initial plan vs. non‐feasible re‐plan are shown in Figs. 2(a) and 2(b), respectively.

### 3.D Mucositis related parameters

**Figure 3:**
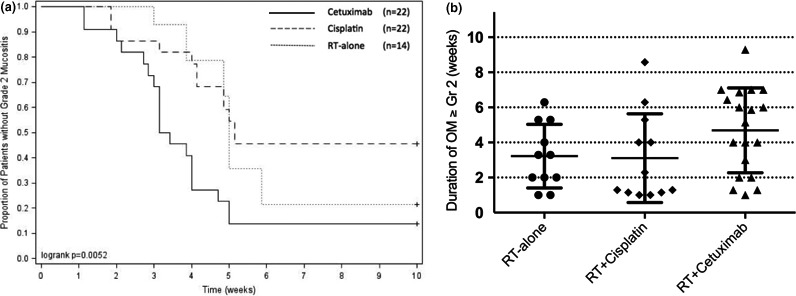
(a) Time‐to‐onset of Gr2 mucositis by treatment group and (b) Duration of Gr2 mucositis in patients treated with IMRT ± cetuximab (The grade of mucositis was assessed from each side of the lateral tongue).

The duration of Gr2 OM was correlated with measured lateral tongue dose (*P* = 0.050). The type of chemotherapy that the patients received was a significant predictor for the time‐to‐onset of Gr2 OM as shown in Fig. 2(a). Patients who underwent IMRT with concurrent cetuximab developed Gr2 OM at a faster rate than those patients who received concurrent cisplatin (*P* = 0.006). Although there was a trend for faster development of Gr2 OM with concurrent cetuximab compared to RT‐alone group, the difference did not reach statistical significance (*P* = 0.086). There was no difference between concurrent cisplatin vs. RT‐alone group (*P* = 0.661) (Fig. 3[a]). As shown in Fig. 3(b), patients receiving concurrent cetuximab tended to experience longer duration of OM ≥Gr2 compared to other patients (*P* = 0.052). Since the difference in duration of OM ≥Gr2 between concurrent cisplatin and RT‐alone group was minimal, we tested the difference between concurrent cetuximab vs. no cetuximab, and concurrent cetuximab was associated with significantly longer duration of OM ≥Gr2 (average duration, 22.5 vs. 10.5 days, *P* = 0.041).

### 3.E Reported pain and quality of life measurement

**Figure 4:**
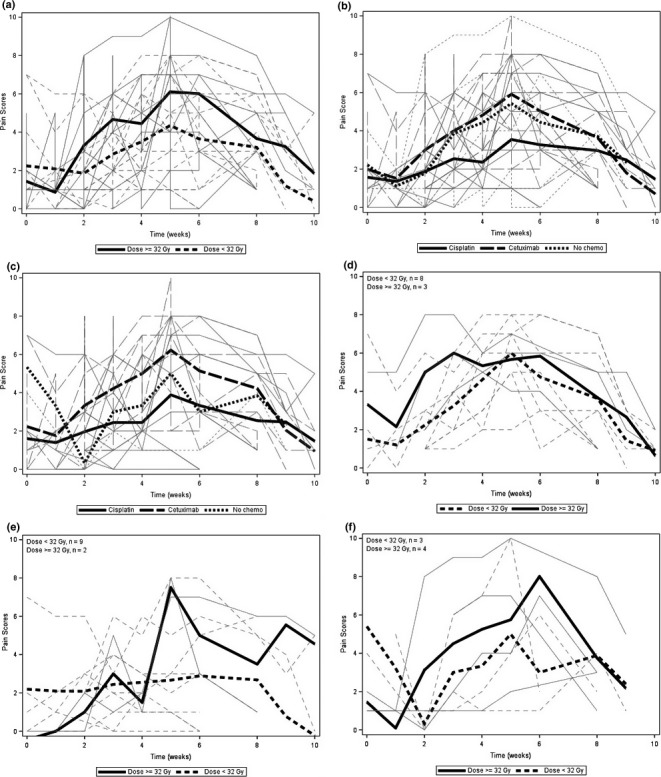
Patient reported mouth pain (a) by measured lateral tongue dose, (b) by treatment type (c) by treatment type after excluding the patients with oral cavity cancer, (d) by dose in patients treated with concurrent cetuximab, (e) by dose in patients treated with concurrent cisplatin, (f) by dose in patients treated with RT‐alone**.**

On the OMWQ‐HN, patients reported severity of their mouth pain on Likert scale from one to ten, with one denoted as “no pain” and ten as “worst imaginable pain.” Although there was a trend for less mouth pain with lower dose with 32 Gy cut‐off (Fig. 4[a]) and concurrent cisplatin (Fig. 4[b]) from week 3 through 7, the difference did not reach statistical significance (low dose, *P* = 0.090 and treatment type, *P* = 0.224). Since the RT‐alone group appeared to have higher pain score than the cisplatin group, we evaluated the tumor site distribution for the three groups. While 57% of the RT‐alone group had oral cavity cancer, only 9% of the cetuximab and 18% of the cisplatin group had tumor located in the oral cavity. Figure 3(c) shows the pain score for the three treatments excluding patients with oral cavity tumor. The scores appear to be similar between the RT‐alone and the cisplatin group.

The interaction of dose, treatment type (cetuximab, cisplatin, and RT only), and treatment time was significant (*P* = 0.005). This means that the pattern over time was different for each treatment type and different depending on the dose level. Figures 4(d)–4(f) show the pain score in relation to RT dose for the different treatment groups, RT and cetuximab, RT and cisplatin, and RT‐alone, respectively. The number of patients in each group became quite small. In general, as expected, lower dose was associated with lower score. Concurrent cetuximab seemed to be related with earlier development of maximum pain, and RT‐alone group reported greatest pain later in their course of treatment (week 3 vs. week 6). RT‐alone group reported relatively higher pain at the beginning of RT compared to other two groups, presumably related to previous surgery. Eighty‐six per centof the RT group received postoperative treatment whereas 18% of the cetuximab and 18% of the cisplatin group received postoperative treatment.

For the two questions assessing global health (GH) and QOL, age >60 for GH (*P* = 0.024), and treatment type for both GH and QOL (*P* = 0.022 and *P* = 0.040 respectively), were significant predictors. When comparing the treatment groups pair‐wise, it was the patients who received cetuximab (vs. RT‐alone) that reported more impairment for both questions assessing GH and QOL (*P* = 0.018 and *P* = 0.032 respectively). In both questions, there was no difference between cetuximab vs. cisplatin (*P* = 0.733 and *P* = 0.580, respectively) nor cisplatin vs. RT‐alone group (*P* = 0.080 and *P* = 0.188, respectively).

## 4 DISCUSSION

To our knowledge, this study is the first to prospectively investigate feasibility of IMRT plan modification based on measured mucosal dose adjacent to dental fillings. OM is a toxicity of radiation and chemotherapy for HNC patients and is associated with pain, functional impairment, treatment interruptions and increased use of costly healthcare resources.^8^, ^9^, ^10^ Dental fillings are a known factor that can exacerbate mucosal toxicity since it generates unpredictable scattering of the radiation and compromises the accuracy of the dose calculation to the adjacent sites due to the imaging artifact that is produced by these fillings on CT scan.^6^, ^7^ Several studies^3^, ^4^, ^5^, ^11^, ^12^, ^13^ have proposed dose and volumetric guidelines to reduce OM by treatment planning intervention but none of them has specifically focused on the effect of dental fillings nor investigated whether it is feasible to optimize the IMRT plan based on such guidelines in vivo. We frequently observe OM on the lateral tongue in patients with dental fillings and this site is also known to be more sensitive to radiation/chemotherapy‐induced mucositis compared to areas of keratinized oral mucosa such as the dorsal tongue, hard palate, and gingiva.^8^ Therefore, we used OSL to measure lateral tongue dose to evaluate the dental filling effect to the adjacent oral mucosa and to see if it is feasible to modify IMRT plan based on the measured doses.

The data collected from this study suggest that it is not feasible to optimize IMRT plan based on measured lateral tongue dose. Compromising target dose was the most frequent reason why we could not adapt the optimized plan. Contrary to the general belief that the metallic dental implants would augment the radiation dose to oral cavity and nearby structures, our study has shown that the influence of dental implants on the dose to the lateral tongue may vary for each patient (Table 3). Possible reasons for the noted disagreement between the measured and calculated doses include a large dose gradient present in oral cavity, a dose variation from positioning uncertainties of the dosimeters, radiation scattering caused by the metallic fillings, and potentially inaccurate calculation on TPS due to streaking artifacts on CT images caused by metals. A number of previous studies have revealed the need for more accurate dose calculation techniques, such as Monte Carlo algorithm, especially when the structure is very close to the metallic implants and the local dose gradient is high, for example the abutting buccal or lingual mucosa as being studied here.^14^, ^15^, ^16^ Novel dose distributions generated by varying beam intensity and geometry within the IMRT software may explain the discrepancy in the trend of dose perturbations between this study and previous studies^17^, ^18^ most of which were done based on conventional RT techniques.

Significant correlation was found between duration of Gr2 mucositis and measured lateral tongue dose. Therefore, further endeavors to find strategies to reduce the lateral tongue mucosal dose without compromising target dose would help reduce such toxicity. Interestingly, the most prominent factor affecting the time‐to‐onset of Gr2 mucositis and duration of mucositis was concurrent cetuximab use. Toxicity and tolerability of cetuximab in treatment of patients with locoregionally advanced head and neck cancer (LAHNC) has been actively studied for years with conflicting results. Bonner et al.,^19^, ^20^ in their prospective randomized trial, compared RT‐alone with RT plus cetuximab and reported improvement in locoregional control and survival with no significant increase in toxicity. Short‐term QOL between the two arms was also similar.^21^ In a retrospective study comparing concurrent cisplatin and RT with cetuximab and RT for LAHNC, concurrent cisplatin achieved better local control and survival but there was no significant difference in late Gr3 or Gr4 effects or feeding tube dependence.^22^, ^23^ However, several other studies reported increased toxicity with concurrent cetuximab compared to RT‐alone or RT plus cisplatin. Pryor et al.^24^ reported higher rate of mucosal toxicity in LAHNC patients treated with concurrent cetuximab compared to Bonner trial, and Walsh et al.^25^ reported significantly higher toxicity including OM in concurrent cetuximab arm compared to concurrent cisplatin arm. A recent randomized phase II trial^26^ also has shown different toxicity profiles between concurrent cisplatin arm vs. concurrent cetuximab arm – hematologic, renal and GI toxicities were more frequent in the cisplatin arm while cutaneous toxicity and need for nutritional support were more frequent in the cetuximab arm – without significant difference in the rate of OM. The results of the large Radiation Therapy Oncology Group 1016 trial with prospective quality of life data collection will hopefully resolve this controversy.

We used OMWQ‐HN to assess GH, QOL, and patient reported mouth pain. In a multicenter longitudinal study, Epstein et al. have shown that OMWQ‐HN is a valid and reliable instrument for assessing the impact of mucositis on patients who are receiving radiation therapy ± chemotherapy for HNC.^27^ It is noteworthy that although concurrent cetuximab significantly affected the onset and the duration of Gr2 OM, it did not reach statistical significance in patient reported pain in OMWQ‐HN, though there was a trend for more mouth pain with concurrent cetuximab from week 3 through 7. This discrepancy reflects the major challenges in defining factors that affect OM and tolerance of patients for OM. Since OM is complex morphologic and functional disorder, variability in assessment methods and inter‐observer differences make it difficult to evaluate the exact incidence and severity of OM and this problem remains unsolved. We used CTCAE v4.0 to assess the changes in morphologic component, and OMWQ‐HN to assess functional component of OM. Interestingly, the pain score reported by RT‐alone group was higher than concurrent cisplatin group and approached close to concurrent cetuximab group (Fig. 3[b]). This is most likely because majority of RT‐alone group had oral cavity cancer, resulting in higher dose to the lateral tongue. RT‐alone group also reported higher pain compared to other treatment groups at the beginning of RT, which is presumably caused by previous surgery (Fig. 3[f]). Therefore, the treatment type and the time point of assessment seem to be the crucial factors to consider when evaluating dose tolerance limits for the oral mucosa. Similar to previous studies,^27^, ^28^, ^29^ items assessing GH and QOL were poorly correlated OM‐related item.

Limitation of this study includes small sample size, short follow up period and lack of additional information that might affect the onset and severity of mucositis, such as comorbidity, social habits^30^ and genetic predispositions.^4^

## 5 CONCLUSIONS

The results of this study show that optimizing IMRT plan based on measured lateral tongue dose is not feasible since such modification tends to compromise the primary aim of curative treatment. Measured lateral tongue dose was associated with longer duration of mucositis and concurrent cetuximab was associated with both earlier onset and longer duration of observed mucositis. The effect of dose on patient reported pain was significantly affected by treatment type and the time‐point in the treatment course. The results of current study should be further validated and improved by studies with larger sample size to establish guidelines and dose constraints for these treatments.

## CONFLICT OF INTERESTS

This work was supported by a grant from Varian Medical System.
